# Predictor of the Simplified Disease Activity Index 50 (SDAI 50) at Month 3 of bDMARD Treatment in Patients with Long-Established Rheumatoid Arthritis

**DOI:** 10.2174/1874312901711010106

**Published:** 2017-09-30

**Authors:** Yusuke Miwa, Mayu Saito, Hidekazu Furuya, Ryo Yanai, Tsuyoshi Kasama

**Affiliations:** 1 Division of Rheumatology, Department of Medicine, Showa University School of Medicine, Tokyo, Japan; 2 Division of Rheumatology, Department of Medicine, Showa University Koto-Toyosu Hospital, Tokyo, Japan

**Keywords:** Rheumatoid arthritis, SDAI 50, Response, bDMARDs, EULAR, SDAI

## Abstract

**Objectives:**

The Simplified Disease Activity Index (SDAI) 50 has good agreement with European League Against Rheumatism (EULAR) response measures for early Rheumatoid Arthritis (RA). There have been reports on early RA, but not on long-established RA. In this study, we analysed the relationships between various baseline factors and SDAI 50 after three months of treatment with biological disease-modifying antirheumatic drugs (bDMARDs) to determine the prognostic factors for long-established RA.

**Methods:**

Subjects were 260 RA patients who had been treated with bDMARDs for 3 months. The following characteristics were investigated: Patient backgrounds, the erythrocyte sedimentation rate (ESR), C-reactive protein and serum matrix metalloproteinase-3 levels, SDAI scores, and health assessment questionnaire disability index and short form-36 scores. As a primary outcome index, the SDAI response was defined as a 50% reduction in the SDAI score between baseline and 3 months (SDAI 50).

**Results:**

Baseline values of disease duration (odds ratio: 0.942, 95% CI: 0.902-0.984), smoking history (odds ratio: 2.272, 1.064-4.850), 28-tender joint count (odds ratio: 0.899, 0.827-0.977), evaluator's global assessment (odds ratio: 1.029, 1.012-1.047) and ESR (odds ratio: 1.015, 1.001-1.030) were determined to be significant factors based on logistic regression analysis.

**Conclusion:**

Our study demonstrated that RA patients with shorter disease duration, no smoking, and higher RA disease activity are more likely to achieve SDAI 50 through bDMARD treatment.

## 1 INTRODUCTION

Recommendations for the treatment of rheumatoid arthritis (RA) are well established [1]. The use of methotrexate (MTX) as an anchor agent, in combination with biological disease-modifying antirheumatic drugs (bDMARDs), has contributed to an increasing number of patients who achieve clinical remission. As a result of this increase in the rate of clinical remission, the number of patients achieving structural and functional remission has also increased [2]. Complete remission in a patient is defined as the achievement of clinical, structural, and functional remission [3].

Predictors of clinical remission in RA have been reported [4, 5]. Current smoking, female sex, longer symptom duration and younger age predict a worse response to MTX in patients with new onset RA [6]. Aiming for Simplified Disease Activity Index (SDAI) [7]remission at month 6 is an appropriate strategy to obtain good functional and structural outcomes at month 12 [8]. Female sex, greater pain, and a lack of initial DMARD therapy reduced the probability of sustained remission [9].

The SDAI 50 shows good agreement compared with the European League Against Rheumatism (EULAR) response measures with early RA. The SDAI 50 response at 3 months appears to be a more significant predictor of outcomes at 6 months than the EULAR response [10]. For example, if the SDAI score improves from 24 to 12, SDAI 12 and 24 reflects moderate disease activity. However, a clinical improvement in the SDAI score from 24 to 12 is beneficial to patients. Compared to EULAR improvement criteria using a disease activity score (DAS) of 28, the SDAI 50 is an index that is easy to calculate and to use in everyday clinical practice. To make the point that SDAI 50 is more sensitive, also show comparable DAS 28 scores where response is not attained. Although multiple studies on clinical remission have been reported, there have been few reports on SDAI 50 response. There have been reports on early RA, but none on long-established RA.

In this study, we analysed the relationships between various baseline factors and SDAI 50 response after three months of bDMARD treatment to determine the prognostic factors in long-established RA.

## 2 MATERIALS AND METHODS

**Fig. (1):**
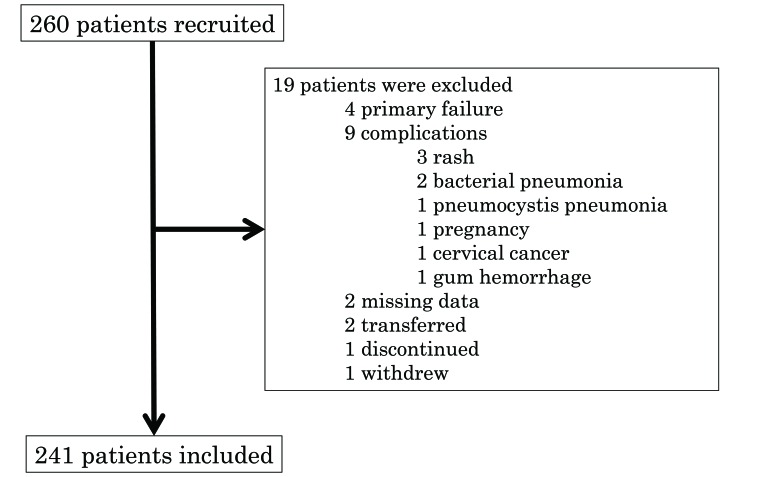
Flow chart of the study.

A retrospective multi-centre study was conducted in the Division of Rheumatology Department of Medicine Showa University Hospital, Showa University Koto-Toyosu Hospital and Showa University Northern Yokohama Hospital. RA patients who initiated bDMARD treatment from 1 January 2007 to 31 March 2016 were examined. Among the 260 patients undergoing bDMARD treatment, 241 were deemed eligible to participate as subjects in this study. Because of missing data, end so on, 19 patients were excluded (Fig. **1**). The bDMARDs used in the study included infliximab for 95 patients, etanercept for 60 patients, adalimumab for 54 patients, golimumab for 28 patients, and certolizumab-pegol for 23 patients. The selection of bDMARDs was left to the primary physician.

The following variables were evaluated at baseline (before the initiation of bDMARD treatment): Age, sex, disease duration, smoking history, body mass index (BMI), experience of bDMARD usage (bio-naïve or bio-switch), steroid dosage, MTX dosage and the combined use of conventional synthetic DMARDs (csDMARDs).

The following variables were evaluated at baseline and after 3 months: 28-swollen joint count (SJC), 28-tender joint count (TJC), patient global assessment (PTGA), evaluator global assessment (PHGA), C-reactive protein (CRP) levels, the erythrocyte sedimentation rate (ESR), and matrix metalloproteinase-3 (MMP-3) levels. Disease activity was evaluated using the SDAI. Activities of daily living (ADL) were evaluated using the health assessment questionnaire disability index (HAQ-DI) [11], and nonspecific health-related quality of life (QOL) was evaluated using the Short Form 36 (SF-36) [12]. The use of adrenocortical steroids (steroids) and non-steroidal anti-inflammatory drugs (NSAIDs) and their dosages before the initiation of bDMARD treatment as well as patient age and disease duration were not exclusion criteria.

As a primary outcome index, SDAI response was defined as a 50% reduction in the SDAI score between the baseline and 3 months (SDAI 50) [10]. To determine the relationship between baseline factors and SDAI 50 outcomes, the baseline values of each item were analysed based on whether SDAI 50 was achieved. The study exclusion criteria included the following: Tocilizumab abatacept and the use of bio-similar compounds; discontinuation of bDMARDs due to primary response failure or adverse effects; additional oral treatment using csDMARD agents or steroids; complications, such as infection; and the likelihood of not continuing the study because of situations such as hospital transfer, patient withdrawal from the study, or other patient circumstances that the primary physician deemed inappropriate for the study. For excluded patients, Last Observation Carried Forward (LOCF) analysis was not performed because of the severe missing data. Although data is available at the time of the start of treatment, the data after the start of treatment is not.

All of the statistical analyses were performed using univariate analyses and multivariate analyses with JMP12 software (SAS Institute Inc., Cary, NC, USA). We obtained written informed consent from all patients who enrolled in the study. The study received approval from the Bio-Ethics Committee of the Department of Medicine, Showa University School of Medicine (No. 1435).

## 3 RESULTS

Two-hundred-sixty patients were included in the study, as shown in Fig. (**1**). Primary failure patients were discontinued within 3 months of the initiation of the treatment and were thus excluded from analysis. In patients with complications, transfers, or discontinuation, the data at the time of discontinuation were missing.

In terms of background parameters for study subjects, 139 patients achieved SDAI 50 (Group A) and 102 did not (Group B) (Table **1**). Based on univariate analyses, Group A had a significantly lower smoking history (p=0.038), higher ESR (p=0.008), SJC (p=0.038), PHGA (p=0.020) and SDAI (p=0.006) than Group B. No other factors showed significant differences between Groups A and B. The findings of multivariate analyses were as follows: Disease duration (odds ratio: 0.942, 95% CI: 0.902-0.984), smoking history (odds ratio: 2.272, 1.064-4.850), TJC (odds ratio: 0.899, 0.827-0.977), PHGA (odds ratio: 1.029, 1.012-1.047) and ESR (odds ratio: 1.015, 1.001-1.030) (Table **2**).

## 4 DISCUSSION

We found that RA patients with lower disease duration, no smoking history, higher TJC, PHGA and ESR at baseline are more likely to achieve SDAI 50 following bDMARD treatment.

In this study, many predictors of RA by bDMARD treatment were reported. Female sex [9, 13-15], older age [14, 16], lower DAS 28 [14, 15, 17], lower SDAI score [8], greater pain [9], lack of initial DMARD [9] and lower HAQ-DI [14, 15] were reported. Pursuing SDAI remission at month 6 is an appropriate strategy to obtain good functional and structural outcomes at month 12 [8].

Unlike SDAI 50, which only reflects a change in disease activity components, the EULAR response includes both disease activity change and a desirable disease activity state achieved, and it was expected to be less inclusive. Compared to EULAR improvement criteria using DAS 28, SDAI 50 is easy-to-calculate and easy-to-use index in daily clinical practice. More EULAR responders (who were SDAI 50 non-responders) were observed at a higher baseline disease activity range than SDAI 50 responders (who were EULAR non-responders), which suggests that the SDAI 50 is a more stringent remission measurement than the EULAR. This report also suggests that in patients with high disease activity, the response to therapy may be underestimated when it is assessed by the SDAI 50 remission measurements, whereas in patients with lower disease activity, the response may be underestimated when using the EULAR response criteria. This possibility must be considered when evaluating the response rate in studies enrolling patients with different levels of disease activity [10]. Although a minority of patients showed discordant response measures at both ends of the disease activity spectrum at baseline, the SDAI 50 response at 3 months appears to be a more significant predictor of outcomes at 6 months than the EULAR response. Although there have been reports on early RA, there are no reports on long-established RA or studies evaluating the predictors of SDAI 50 response using bDMARDs.

These data suggest that patients with high disease activity achieved SDAI 50 response measurements. The SDAI 50 response measurements at 3 months are more significantly associated with low disease activity score/remission at 6 months than are the EULAR remission measurements, implying that although it is less sensitive than the EULAR response at high disease activity, once it is achieved, the likelihood of achieving the target outcome is greater with the use of the SDAI 50 response measurements [10]. Clinicians tend to rely more on disease states than response measures for clinical decision-making in standard practice. For example, if the SDAI score improves from 24 to 12, SDAI 12 and 24 represent moderate disease activity. However, clinically improving from SDAI 24 to 12 is beneficial to patients.

Our study had some limitations. First, we did not perform a radiographic evaluation of the joints, although we are aware that a radiographic evaluation is expected to influence the Damage-HAQ-DI [18]. Although HAQ-DI contributed to functional remission according to a multiple logistic regression analysis, the modified total vdH-Sharp score did not play a role during abatacept treatment in RA [8, 19]. In addition, because radiographic evaluations were only performed in 50 patients, the subjects were analysed without the use of radiographic evaluations. Second, we used actual clinical data that accumulated over a long period, which generated a bias in the biologicals used in this study; therefore, the clinical outcomes may differ depending on the type of biological treatment. Third, the study design is not a prospective study but a retrospective one. Finally, no socioeconomic factors were included in our analysis.

## CONCLUSION

In conclusion, our study demonstrated that RA patients with shorter disease duration, no smoking, and higher disease activity of RA are more likely to achieve SDAI 50 through bDMARD treatment.

## Figures and Tables

**Table 1:** Univariate analysis of the demographics and baseline characteristics of 241 RA patients.

		Achieved SDAI 50	Did not achieve SDAI 50	P
		(Group A)	(Group B)	
n		139	102	
Age (years)		60 (43-68)	57 (46-68)	0.946*
Sex (female, %)		82	85	0.616**
Disease duration (year)		4 (2-9)	4 (1-11)	0.370*
Smoking history (yes, %)		22	35	0.038**
Body mass index		21 (20-24)	21 (19-24)	0.537*
Biological agent (naïve, %)		78	68	0.083**
Steroid dosage (mg/d)		2 (0-5)	2 (0-5)	0.905*
MTX dosage (mg/w)		8 (6-10)	8 (6-12)	0.105*
Combined csDAMRDs (yes, %)		17	16	0.880**
ESR (mm/H)		38 (16-67)	21 (12-51)	0.008*
CRP (mg/dL)		1 (0.29-3.74)	1 (0.143-2.643)	0.053*
RF (mg/dL)		64 (18.825-197.95) n=134	56 '26.35-135.15) n=99	0.381*
MMP-3 (ng/mL)		150 (74.9-312.4) n=132	140 (64-309) n=100	0.979*
TJC		7 (4-11)	6 (2.25-10)	0.178*
SJC		4 (2-8)	3 (1-6)	0.038*
PTGA		53 (31-74.5)	55 (28-70.75)	0.519*
PHGA		65 (32-76)	48 (22.25-72)	0.020*
SDAI		26 (17-35)	18 (11-28) n=126	0.006*
HAQ-DI		0 (0.125-1) n=137	1 (0.09375-1.125) n=96	0.281*
SF-36	PCS	30 (21-39) n=103	30 (21-40) n=80	0.682*
	MCS	50 (44-54) n=103	49 (44-55) n=80	0.791*
	RCS	45 (31-56) n=103	45 (32-57) n=80	0.824*

MTX, methotrexate

csDMARD, conventional synthetic disease-modifying antirheumatic drugs

RF, rheumatoid factor

MMP-3, matrix metalloproteinase 3

SDAI, simplified disease activity index

TJC, tender joint count

SJC, swollen joint count

PTGA, patient’s global assessment

PHGA, evaluator's global assessment

HAQ-DI, health assessment questionnaire disability index

SF-36, short form-36.

PCS, physical component summary score

MCS, mental component summary score

RCS, role/social component summary score

*analysis using MannWhitney U test

**analysis using chi-squared test for independence

**Table 2:** Prognostic factors identified by multivariate analysis showing a significant association with SDAI 50.

	Achieved SDAI 50		Did not achieve SDAI 50		Odds ratio (95% CI)		P
Disease duration (year)	4 (2-9)		4 (1-11)		0.942 (0.902-0.984)		0.0069
Smoking history (yes, %)	22		35		2.272 (1.064-4.850)		0.0339
TJC	7 (4-11)		6 (2.25-10)		0.899 (0.827-0.977)		0.0121
PHGA	65 (32-76)		48 (22.25-72)		1.029 (1.012-1.047))		0.0009
ESR (mm/H)	38 (16-67)		21 (12-51)		1.015 (1.001-1.030)		0.0369

TJC, tender joint count

PHGA, evaluator's global assessment

ESR, erythrocyte sedimentation rate
